# Durability-enhanced two-dimensional hole gas of C-H diamond surface for complementary power inverter applications

**DOI:** 10.1038/srep42368

**Published:** 2017-02-20

**Authors:** Hiroshi Kawarada, Tetsuya Yamada, Dechen Xu, Hidetoshi Tsuboi, Yuya Kitabayashi, Daisuke Matsumura, Masanobu Shibata, Takuya Kudo, Masafumi Inaba, Atsushi Hiraiwa

**Affiliations:** 1 Faculty of Science and Engineering, Waseda University, 3-4-1, Ohkubo, Shinjuku-ku, Tokyo 169-8555, Japan; 2 The Kagami Memorial Laboratory for Materials Science and Technology, Waseda University, 2-8-26 Nishiwaseda, Shinjuku, Tokyo 169-0051, Japan; 3 Research Organization for Nano & Life Innovation, Waseda University, 513 Waseda-tsurumaki, Shinjuku, Tokyo 162-0041, Japan

## Abstract

Complementary power field effect transistors (FETs) based on wide bandgap materials not only provide high-voltage switching capability with the reduction of on-resistance and switching losses, but also enable a smart inverter system by the dramatic simplification of external circuits. However, p-channel power FETs with equivalent performance to those of n-channel FETs are not obtained in any wide bandgap material other than diamond. Here we show that a breakdown voltage of more than 1600 V has been obtained in a diamond metal-oxide-semiconductor (MOS) FET with a p-channel based on a two-dimensional hole gas (2DHG). Atomic layer deposited (ALD) Al_2_O_3_ induces the 2DHG ubiquitously on a hydrogen-terminated (C-H) diamond surface and also acts as both gate insulator and passivation layer. The high voltage performance is equivalent to that of state-of-the-art SiC planar n-channel FETs and AlGaN/GaN FETs. The drain current density in the on-state is also comparable to that of these two FETs with similar device size and *V*_*B*_.

In an ideal inverter system (Fig. 1) for next-generation power conversion systems, a combination of n-channel and p-channel power field-effect transistors (FETs) with equivalent power switching performance is required to achieve complementary operation for power compact systems such as simple gate drive circuits. Diamond, in addition to having the highest reported breakdown field and thermal conductivity, also has the highest hole mobility^1^, the highest p-type conductivity^2^,^3^ and a two-dimensional hole gas (2DHG)^4^ that makes it a unique and fascinating p-channel material for complementary inverter applications (Fig. 1). In wide bandgap semiconductors, such as III-nitrides, p-type conductivity is much less prevalent than n-type conductivity. The superior p-type nature of diamond enables its use in p-channel FETs; the highest drain current density was reported in wide bandgap p-channel FETs at more than 1 A/mm^5^ and an on-resistance of 4 Ωmm at a submicron gate length that is comparable to that of n-channel AlGaN/GaN FETs^6^, because the surface density of the 2DHG is as high as 10^12^,^13^ cm^−2  ^7,8. The 2DHG appears where there is a hydrogen-terminated (C-H) diamond surface that has appropriate adsorbates^9^,^10^ or film coatings^9^ to induce holes (Supplementary Fig. S1).

The reliability of C-H diamond FETs has been improved by the passivation^11^,^12^ of the adsorbates on C-H diamond by Al_2_O_3_ atomic layer deposition (ALD). Recently, the high-temperature stability of a 2DHG^13^ was reported after ALD of Al_2_O_3_ at 450 °C on a cleaned C-H diamond surface by *in situ* removal of the adsorbates immediately before the ALD process. The 2DHG is generated by the ALD Al_2_O_3_ itself, and not necessarily by the adsorbates. The ALD Al_2_O_3_ film induces hole accumulation on the n-type Si (n-Si) surface, i.e., a p-type inversion layer^14^ is produced by the negative charging (electron occupation) of Al_2_O_3_ near its interface with n-Si. At the C-H diamond surface, hole accumulation is caused by the negative charging of unoccupied levels such as the interstitial oxygen point defects O_i_^15^,^16^ or the aluminium vacancies V_AL_^15^. These levels are located below the C-H diamond valence band edge, as shown in Fig. 2a. The Oi level (unoccupied states) is located 1.0 eV^16^ above the valence band edge and the valence band offsets between C-H diamond and Al_2_O_3_ are 2.9 eV^17^ and 3.9 eV^18^, respectively. When the Oi states near the interface are occupied by electrons with a density of more than 10^12^ cm^−2^, 2DHG formation with an equivalent hole density near the interface in C-H diamond satisfies the charge neutrality condition.

Using the high temperature (450 °C) ALD Al_2_O_3_ as gate oxide and passivation of gate-drain region (drift region), a C-H diamond metal oxide semiconductor (MOS) FET was uniquely designed for high-voltage and high-temperature operation. It is shown schematically as a cross-sectional structure in Fig. 2b and as a 3D image in Fig. 2c. The MOSFET shows clear pinch-off and saturation characteristics with a high on-off ratio in the temperature range from −263 °C (10 K) to 400 °C (673 K) (Supplementary Figs S2 and S3).

We consider two typical MOSFETs, which both have Al_2_O_3_ gates; the first has a passivation oxide thickness of 200 nm, a gate length (*L*_*G*_) of 2 μm, a source-gate distance (*L*_*SG*_) of 2 μm and a gate-drain distance (*L*_*GD*_) of 17 μm, while the second has an oxide thickness of 400 nm, an *L*_*G*_ of 9 μm, an *L*_*SG*_ of 3 μm and an *L*_*GD*_ of 16 μm, and their maximum drain current densities (*I*_*DS*_) were 116 mA/mm and 110 mA/mm, according to their *I*_*DS*_-*V*_*DS*_ characteristics (Fig. 3a and c), respectively. The two MOSFETs exhibited breakdown voltages (*V*_*B*_) of 1538 V and 1662 V (Fig. 4a, Fig. 5), respectively. Using a device simulation based on the two-dimensional negatively charged sheet model (Fig. 2a, Fig. 6a, Supplementary Fig. S4), the *I*_*DS*_-*V*_*DS*_ characteristics shown in Fig. 3a were reproduced almost exactly in terms of their *V*_*GS*_ dependence in Fig. 3b using a negative charge areal density (*N*_*S*_) of 5 × 10^12^ e cm^−2^ with a hole channel mobility of 80 cm^2^V^−1^ s^−1^ at the Al_2_O_3_/C-H diamond interface. In general, the charge sheet model is successful in reproducing the characteristics of an AlGaN/GaN FET, where the interface polarization produces the positive charge sheet that is responsible for the two-dimensional electron gas. At the Al_2_O_3_/C-H diamond interface, a 2DHG might be formed by the negatively charged sites near the interface as shown in Fig. 2a.

The off-state characteristics before breakdown of the diamond MOSFETs of Al_2_O_3_ (mostly 200 nm in thickness) with a common gate width (25 μm) and different gate-drain length (*L*_*GD*_) values of 9, 16, 17, and 22 μm were investigated at room temperature (RT) and at higher temperatures until breakdown started to occur, and the results are shown in Fig. 4. *V*_*GS*_ for the off-states are +20- + 60 V. The maximum *V*_*B*_ at each *L*_*GD*_ are 996 V (*L*_*GD*_ = 9 μm), 1270 V (20 μm, 300 °C), 1512 V (17 μm, 200 °C), 1646 V (22 μm), and 1662 V (16 μm, with a thicker oxide layer of 400 nm). The source-drain current (drain leakage current) *I*_*DS*_ gradually increases at higher temperatures. At 800 V, the leakage currents at room temperature range from 2 × 10^−7^ to 5 × 10^−6^ A/mm (Fig. 4a) and the corresponding values at 200 and 300 °C are 8 × 10^−6^ A/mm and 10^−3^ A/mm (Fig. 4b), respectively. In general, the drain leakage current increases gradually at a high voltage (high electric field) and breakdown starts to occur. These values are acceptable for power device applications. In contrast, the source-gate-drain current (or gate leakage current) *I*_*DGS*_ is one to two orders of magnitude less than *I*_*DS*_, as shown in Fig. 4. At the start of the breakdown process, when *I_DGS_* reaches *I_DS_*, gate-drain breakdown through the gate oxide may be a main cause. One such example is a FET with *L*_*GD*_ = 9 μm and *V*_*B*_ = 996 V (see Fig. 4a). In other cases, however, *I*_*DGS*_ is less than *I*_*DS*_ by more than an order of magnitude at breakdown. Most breakdown processes are initiated at the drift region near the gate.

The maximum breakdown voltage *V*_*B*_ at each *L*_*GD*_ is shown in Fig. 5 with two different passivation thicknesses (200 nm and 400 nm). When *L*_*GD*_ is 1 μm, *V*_*B*_ = 365 V is obtained (Supplementary Fig. S5). For a short *L*_*GD*_ of 1 μm, the punch-through condition (under which the difference between the electric fields along the Al_2_O_3_ /C-H diamond interface on the gate and drain sides is small) has been applied based on the electric field distribution that can be calculated via device simulations, as discussed later in this work. The averaged electric field is simply calculated to be *V*_*B*_/*L*_*GD*_ = 3.6 MV/cm. This value is equivalent to that of the breakdown fields of SiC and GaN. As *L*_*GD*_ increases to 9 μm, *V*_*B*_ also increases and reaches 996 V at *L*_*GD*_ = 9μm. As *L*_*GD*_ increases further, *V*_*B*_ also increases correspondingly until *L*_*GD*_ = 22 μm. *V*_*B*_ values of 1600 V and 1646 V are obtained at *L*_*GD*_ = 20 and 22 μm (Fig. 5), respectively. With a thicker (400 nm) Al_2_O_3_ passivation layer between the gate and the drain, *V*_*B*_ values from 1097 to 1708 V are obtained from *L*_*GD*_ = 11 to 16 μm (Fig. 5). When a thicker oxide layer is used, *V*_*B*_ increases above 1000 V up to 1700 V with increasing *L*_*GD*_. The peak electric field at diamond side with thicker passivation layer (400 nm) is decreased by 25–30% compared with that of 200 nm thickness (discussed later). The *V*_*B*_ of 1708 V (Supplementary Fig. S6) is the highest value ever reported for a diamond FET with on-state *I*_*DS*_~100 mA/mm, and is comparable to that of SiC^19^ or AlGaN/GaN^20^,^21^ FETs with similar *L*_*GD*_ (Table 1).

In the off-state of the MOSFET with the two-dimensional negative charge sheet (Fig. 2a, Fig. 6a), the electric field along the Al_2_O_3_/C-H diamond interface in the lateral direction contains a peak that occurs near the gate edge, as indicated by the cross shown in Fig. 2b, with a maximum value denoted by *E*_*M*_. The electric field along the Al_2_O_3_/diamond interface decreases with increasing distance from the gate edge, and almost reaches zero at a distance *L*_*0*_ from the gate edge. The electric field distribution along the Al_2_O_3_/diamond interface is evaluated using MOSFET device simulations with various densities (*N*_*S*_) of the 2D negative charges (1 × 10^11^ × 10^13^ cm^−2^) that were distributed homogeneously at the Al_2_O_3_/C-H diamond interface (Fig. 6a). In Fig. 6b and c, along the Al_2_O_3_/diamond interface, several of the electric field distributions that are responsible for *V*_*DS*_ = 1600 V are shown for various *N*_*S*_. The reason why *V*_*DS*_ = 1600 V was selected as an example here is that it is approximately equal to the frequently observed value of the highest operating voltage that is available in FETs at present. The *V_GS_* for the off-state is maintained at 30 V, so the gate-drain voltage drop is higher than the source-drain voltage drop. However, the gate potential does not have a major effect on the electric field or the potential distribution between the source and the drain. *E*_*M*_ is effectively reduced by the thicker Al_2_O_3_ (400-nm-thick) layer at each *N*_*S*_. At *N*_*S*_ = 5 × 10^12^ cm^−2^, which reproduces an experimental *I*_*DS*_*-V*_*DS*_ characteristic in the on-state (Fig. 3a and b), the *E*_*M*_ and *L*_*0*_ values with the 200-nm-thick Al_2_O_3_ layer are calculated to be 8.1 MV/cm and 3.5 μm at *V*_*DS*_ = 1600 V (Fig. 6b), respectively. The corresponding values when using a 400-nm-thick Al_2_O_3_ layer are calculated to be 5.9 MV/cm and 4.5 μm at 1600 V, respectively (Fig. 6c). Because the voltage drop of 1600 V occurs within the calculated *L*_*0*_ (<5 μm), MOSFETs with *V*_*B*_ ~1600 V are expected to be realized at an *L*_*GD*_ of just over 5 μm, but are actually obtained experimentally at *L*_*GD*_ > 16 μm (Fig. 5). This inconsistent result indicates that that the real value of *L*_*0*_ is greater and the real *E*_*M*_ is smaller than the values calculated based on *N*_*S*_ = 5 × 10^12^ cm^−2^. It is reasonable that the effective value of *N*_*S*_ is much lower than 5 × 10^12^ cm^−2^. At *N*_*S*_ = 3 × 10^11^ cm^−2^, *L*_*0*_ reaches 16 μm, as shown in Fig. 6b and c. The fact that *V*_*B*_ ~1600 V is obtained at an *L*_*GD*_ of more than 16 μm indicates that *N*_*S*_ < 3 × 10^11^ cm^−2^. As shown in Fig. 5, the maximum value of *V*_*B*_ is roughly proportional to *L*_*GD*_. This behaviour may also be caused by a lower effective charge density, such as *N*_*S*_ < 3 × 10^11^ cm^−2^.

The original negative charge density can be either reduced or cancelled in one of two main ways. The first involves hole injection into the Al_2_O_3_ layer on an undoped diamond layer. Energetic (hot) holes that are accelerated by a high electric field can enter the Al_2_O_3_ layer beyond the large band offset between the Al_2_O_3_ layer and C-H diamond (2.9–3.9 eV) (Fig. 2a)^17^,^18^. This effect on the electric field distribution can also be simulated using smaller *N*_*S*_ values such as *N*_*S*_ = 1 × 10^12^ and 3 × 10^11^ cm^−2^, as shown in Fig. 6b and c. The other way involves use of positively ionized nitrogen donors in a nitrogen-doped diamond substrate (nitrogen concentration of 10^19^ cm^−3^) under the undoped diamond layer (Fig. 2b). Substitutional nitrogen atoms that act as deep donors can be positively ionized via hole recombination with an electron from a neutral nitrogen donor. The deep donor level (1.7 eV) means that this positive charge can be maintained for a long time because of the very low density of the conduction electrons. The ionized donors are randomly distributed in a nitrogen-doped substrate beneath the undoped layer. For simplicity, we assume here that the donors are distributed as a positive charge sheet with a charge areal density of *N*_*B*_. In the device simulations, it is varied from 1–6 × 10^12^ cm^−2^ at the bottom of the undoped diamond (Fig. 6a) and *N*_*S*_ is fixed at 5 × 10^12^ cm^−2^ (*N*_*S0*_), then *N*_*B*_ compensates *N*_*S0*_ to produce *N*_*S0*_ − *N*_*B*_. When *N*_*S0*_ − *N*_*B*_ produces values of 1 × 10^12^ and 3 × 10^11^ cm^−2^, the corresponding electric field distributions (which are not shown here) are calculated to be almost the same as those shown at *N*_*S*_ = 1 × 10^12^ and 3 × 10^11^ cm^−2^ in Fig. 6b and c. When *N*_*S0*_ − *N*_*B*_ = 0, perfect charge compensation occurs and the electric field distribution becomes flat, as shown in Fig. 5b and c (indicated by the green lines). This is an ideal situation for high-voltage device structures such as super-junctions. When 1 × 10^12^ cm^−2^ > *N*_*S0*_ − *N*_*B*_ > −1 × 10^12^ cm^−2^. the maximum *I*_*DS*_ is preserved at 60–90% of the value obtained by the *N*_*S0*_ (Fig. 3b), indicating that the positively charged embedded layer does not reduce the channel and drift hole conduction so greatly near the surface.

The drain current density (*I*_*DS*_) of the diamond FET with the breakdown voltage of 1538 V and 1662 V exceeds 100 mA/mm, according to the *I*_*DS*_*-V*_*DS*_ characteristics at low drain bias shown in Fig. 3. Saturation behaviour is observed at *V*_*GS*_ > 0 V and pinch-off is obtained at *V*_*GS*_ = 50 V. At *V*_*GS*_ < 0 V, the drain current increases and becomes linear. Finally, the drain current does not increase further upon application of a more negative gate bias. The saturated slope of the *I*_*DS*_-*V*_*DS*_ characteristic is mainly due to the gate-drain resistance, because it is the main resistive part of the high voltage device. However, the total drain on-current for a source-to-drain distance of 25–30 μm is more than 100 mA/mm, which is comparable to the corresponding currents of SiC lateral MOSFETs (90 mA/mm)^19^, AlGaN/GaN HFETs (300–600 mA/mm)^20^,^21^ and AlGaN/AlGaN HFETs (200 mA/mm)^22^ with an equivalent device size and *V*_*B*_ (Table 1). A higher on-current can be achieved at a lower *V*_*DS*_ by increasing either the hole areal density or the sub-surface mobility.

The maximum *I*_*DS*_ was investigated as a function of temperature for diamond FETs with the C-H channel (2DHG) and with a boron-doped channel (Fig. 7). The *I*_*DS*_ of the C-H channel only changes by approximately 40% from −263 °C to 300 °C. In the C-H channel MOSFETs, which show *V*_*B*_ of more than 1000 V, *I*_*DS*_ is approximately 20 mA/mm at *V*_*DS*_ = 10 V and approximately 100 mA/mm at *V*_*DS*_ = 50 V at room temperature. At higher temperatures, *I*_*DS*_ decreases slightly because of the reduction in hole mobility caused by phonon scattering. In contrast, the boron-doped channel FETs with a high *V*_*B*_ of more than 600 V show a rapid increase in *I*_*DS*_ by more than one order of magnitude over the range from room temperature to 200–300 °C. This is caused by the activation of boron as a relatively deep acceptor (0.37 eV), with doping levels of 5 × 10^15^ and 1 × 10^17^ cm^−3^ in the metal-semiconductor field-effect transistor (MESFET)^23^ and the junction FET (JFET)^24^,^25^, respectively. The drain current densities of the 2DHG channel are more than two orders and one order of magnitude higher than those of the boron-doped channel at room temperature and in 200–300 °C range, respectively.

Junction FETs, including MESFETs, regulate their depletion layer fronts far away from their p-n or MES junctions to control their bulk p-type channel thicknesses. Therefore, the drain current controllability in these FETs is low, but the current is stable because the channel carriers are not affected by the junction charge. Because a MOSFET can control surface band bending, the current response of its gate is efficient, but it is also sensitive to interface charge. The 2DHG that occurs at the Al_2_O_3_/C-H diamond interface may originate from the electric field (band bending) that is induced by the negative charges in Al_2_O_3_ near the interface and the applied gate voltage. The drain current can be increased or reduced effectively, because the C-H surface has a very low surface state density when compared with that of the C-O surface that is normally used in MESFETs.

High performance 2DHG diamond MOSFETs with submicron gate lengths have already obtained maximum current densities of 1.2–1.3 A/mm^5^,^26^, on-resistances of 4–6 Ωmm^5^, and transconductances of 200–480 mS/mm^5^. These values are comparable to those of AlGaN/GaN devices of the same size. In the long *L*_*GD*_ diamond FETs that are used for high voltage applications, however, the drain current density and the transconductance become more than one order of magnitude lower because of the low mobility (70–100 cm^2 ^V^−1^ s^−1^) in the 2DHG drift layer. Mobility enhancement of the 2DHG layer by up to 500 cm^2^ V^−1 ^s^−1^ or the discovery of a shallow acceptor with an acceptor level that is lower than 0.2 eV will dramatically improve the specific on-resistance for high voltage applications.

## Conclusions

By creation of a 2DHG using a high-temperature ALD Al_2_O_3_ layer on a C-H bond diamond surface, the following p-channel MOSFET characteristics have been obtained.

1) Off-state. When *L*_*GD*_ increases from 1 μm to 22 μm, *V*_*B*_ increases up to roughly 1600 V in the off-state with the increasing *L*_*GD*_. The maximum breakdown voltage is 1700 V at room temperature, 1500 V at 200 °C and 1200 V at 300 °C and 400 °C.

2) On-state. In the high voltage FET with *V*_*B*_ of 1600 V, the maximum current density in the on-state when normalized with respect to the channel width is 100 mA/mm and is nearly constant from −200 °C (73 K) to 400 °C (673 K).

**Figure 1:**
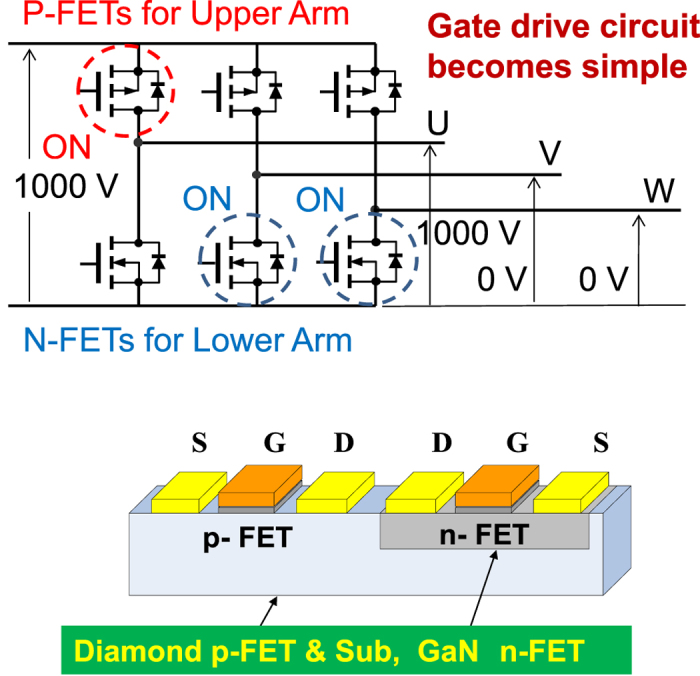
System of complementary wide bandgap semiconductor devices such as that used for power inverters, where the source potentials of the n-channel FETs in the lower arm and the p-channel FETs in the upper arm are fixed at ground level and at a high level, respectively. This is an almost ideal circuit and is much simpler than that used in current inverter circuits, in which n-channel FETs are used in both the upper and lower arms, and the source potential in the upper arm is not fixed and thus must be changed by on-off switching. An extra gate drive circuit is needed in the current inverter circuit to apply the appropriate gate-source voltage, but is not required in the complementary system shown.

These high-voltage breakdown characteristics in the off-state and the drain current density per unit gate width in the on-state are comparable to those characteristics of lateral SiC and III-nitride FETs. Because the 2DHG at the C-H diamond surface is ubiquitous and was stably covered with ALD Al_2_O_3_, the above FET performance can easily be transferred to vertical power devices such as trench gate MOSFETs. These results demonstrates the potential for application of a diamond p channel FET as a smart power inverter using complementary power FETs (Fig.1).

## Method

**Figure 2:**
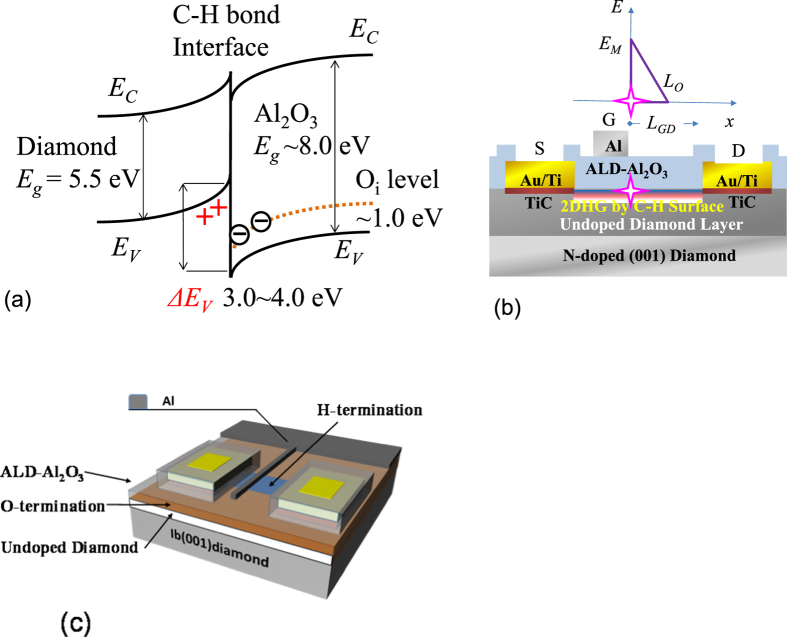
(**a**) Energy band diagram of interface between Al_2_O_3_ and H-terminated (C-H) diamond. The band offset at the valence band edge is 3.0–4.0 eV^17^,^18^. Oi is the unoccupied energy level of the interstitial oxygen located at 1.0 eV from the valence band edge of Al_2_O_3_. Oi is negatively charged and holes thus accumulate at the C-H diamond surface. (**b**) Cross-sectional and (**c**) 3D structure representations of C-H diamond FET with 200-nm-thick Al_2_O_3_ gate insulator and drift passivation layer with an asymmetric source and drain structure, where the gate-drain distance *L*_*GD*_ was varied from 1 μm up to 25 μm. Electric field distribution along the Al_2_O_3_/C-H diamond interface is schematically shown in (**b**), where a cross symbol indicates the point of the highest electric field on the diamond side.

The C-H diamond MOSFET for high voltage and high temperature operation is shown schematically as a cross-sectional structure in Fig. 2b and as a 3D image in Fig. 2c. The MOSFET fabrication process without deterioration of the C-H bonds is briefly described in the following. The starting substrate is synthetic diamond (001), which was formed under high-pressure and high-temperature (HPHT) conditions. In this HPHT synthetic diamond, nitrogen atoms are incorporated as deep donors at a concentration of 10^19^ cm^−3^. On this substrate, a nominally undoped diamond layer is homoepitaxially grown by microwave plasma-assisted chemical vapour deposition to a thickness of 0.5 μm (Supplementary Fig. S7a). Source and drain metal contacts are then formed by Au/Ti deposition (Supplementary Fig. S7b). Most of the surface area, except for the source and drain contacts, is then H-terminated by remote plasma treatment at 600 °C (Supplementary Fig. S7c). During that time, TiC is formed to erode the diamond layer to a depth of a few nm and subsequently form a stable contact to the diamond. The H-terminations are replaced with O-terminations through a local oxidation process to form an isolated region, and thus an H-terminated channel remains between the source and drain (Supplementary Fig.S7d). Al_2_O_3_ is then deposited at 450 °C by ALD to simultaneously form the gate insulator and the passivation layer (Supplementary Fig. S7e). During Al_2_O_3_ formation, the conditions required for the 2DHG are satisfied. Finally, the gate metal (Al) is deposited and the gate is patterned using a lift-off process (Fig. 2c, Supplementary Fig. S7f).

## Additional Information

**How to cite this article**: Kawarada, H. *et al*. Durability-enhanced two-dimensional hole gas of C-H diamond surface for complementary power inverter applications. *Sci. Rep.* **7**, 42368; doi: 10.1038/srep42368 (2017).

**Publisher's note:** Springer Nature remains neutral with regard to jurisdictional claims in published maps and institutional affiliations.

## Supplementary Material

Supplementary Information

## Figures and Tables

**Figure 3:**
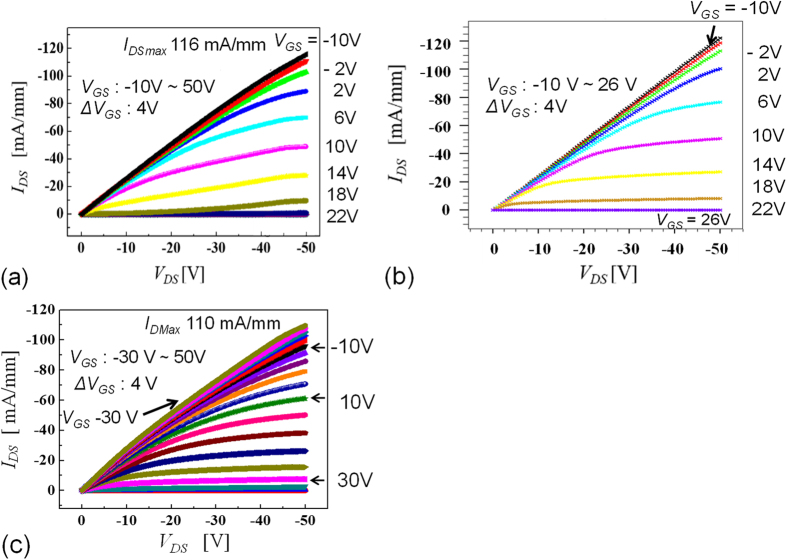
*I*_*DS*_*-V*_*DS*_ characteristics at low drain bias and various gate voltages for a diamond FET with breakdown voltages of 1538 V and 1662 V. Saturation behaviour is observed at *V*_*GS*_ > 0 V and pinch-off is obtained at *V*_*GS*_ = 30 V. (**a**) *L*_*G*_ = 2 μm, *L*_*GD*_ = 17 μm, and oxide thickness of 200 nm, (**b**) Simulation of (**a**); (**c**) *L*_*G*_ = 9 μm, *L*_*GD*_ = 16 μm, and oxide thickness of 400 nm.

**Figure 4:**
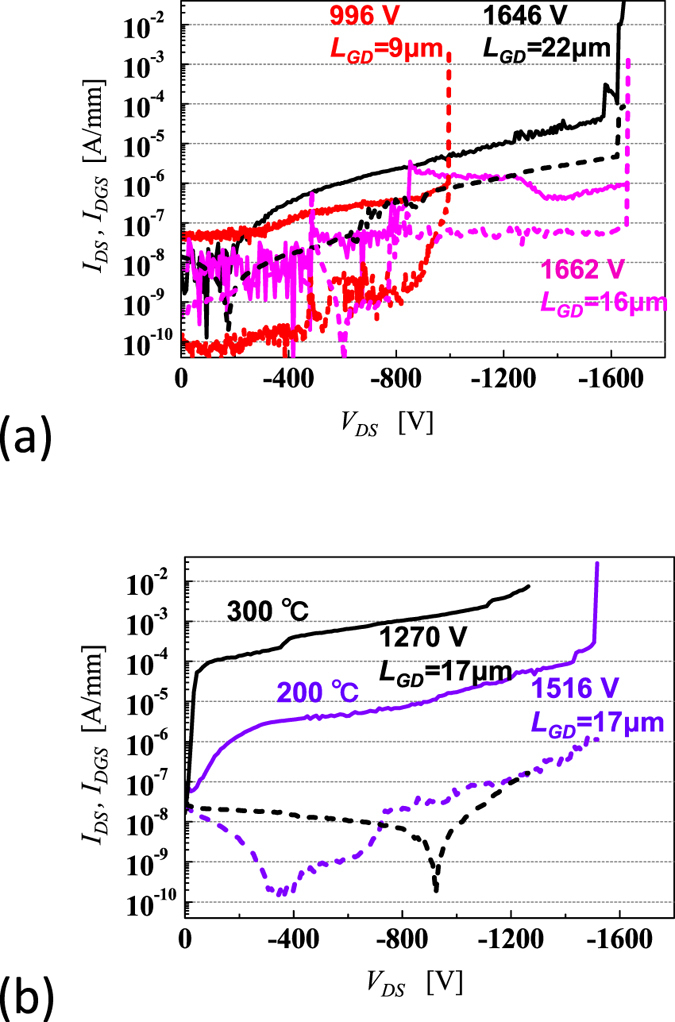
Logarithmic *I*_*D*_*-V*_*DS*_ characteristics showing the blocking behaviour of C-H diamond MOSFETs with a common gate width (25 μm) in the off-state for various *L*_*GD*_ values of 9, 16, 17, 20 and 22 μm. Solid lines represent the source-drain current (*I*_*DS*_) and dotted lines represent the source-gate-drain current (*I*_*DGS*_).(**a**) For *L*_*GD*_ values of 9, 16, and 22 μm, the breakdown voltages are *V*_*B*_ = 996, 1662, and 1646 V at room temperature, respectively. (**b**) For *L*_*GD*_ of 17 μm, the breakdown voltages are *V*_*B*_ = 1516 and 1270 V at 200 °C and 300 °C, respectively.

**Figure 5:**
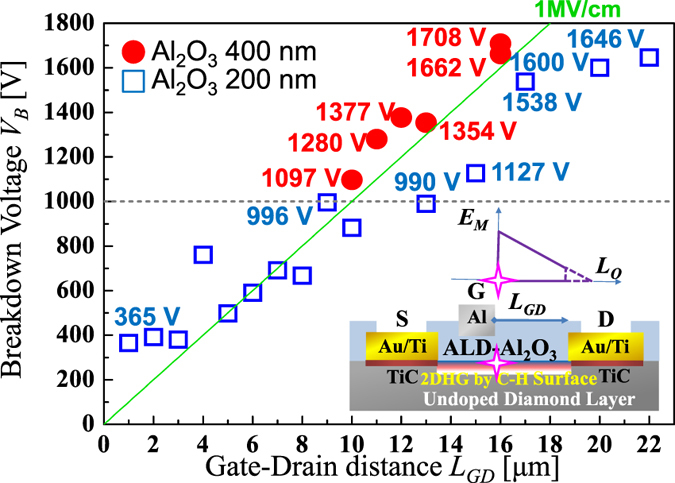
Maximum breakdown voltage (*V_B_*) as a function of gate-drain length (*L_GD_*). The MOSFETs are composed of a 200-nm-thick Al_2_O_3_ layer as the gate insulator and 200- or 400-nm-thick Al_2_O_3_ layers acting as a passivation layer between the gate metal and drain metal. *V*_*GS*_ for the off-states are +20- + 60 V.

**Figure 6:**
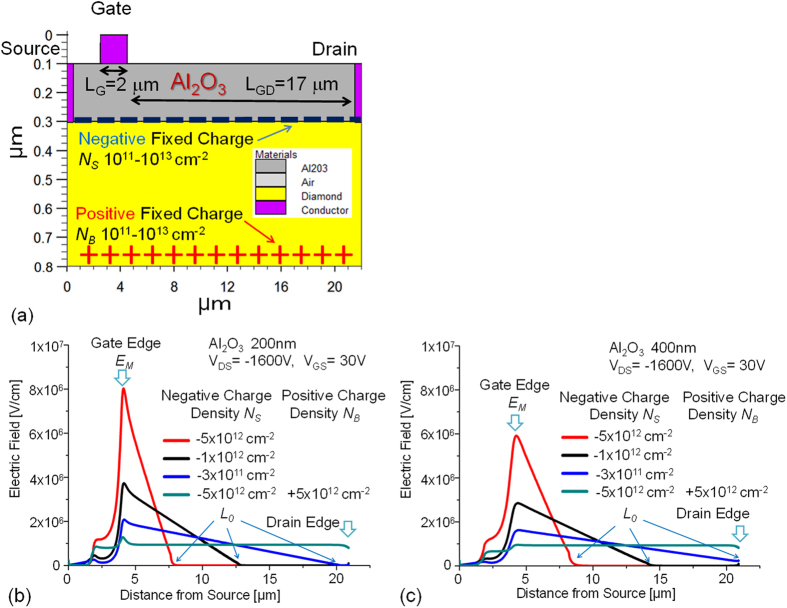
(**a**) Simulation model for a MOSFET displaying the *I*_*DS*_-*V*_*DS*_ characteristics shown in Fig. 3a. A MOS structure is composed of metal, Al_2_O_3_ and undoped diamond. Source and drain metals contact with the undoped diamond at both edges with 0 eV Schottky barrier height. Negative and positive charge sheets are spaced at the Al_2_O_3_/diamond interface and at the bottom of undoped diamond, respectively. (**b**) Electric field distributions along the Al_2_O_3_/C-H diamond interface that were calculated using the model shown in (**a**), where the Al_2_O_3_ thickness is 200 nm. (**c**) The same electric field distributions, calculated when the Al_2_O_3_ thickness is 400 nm.

**Figure 7:**
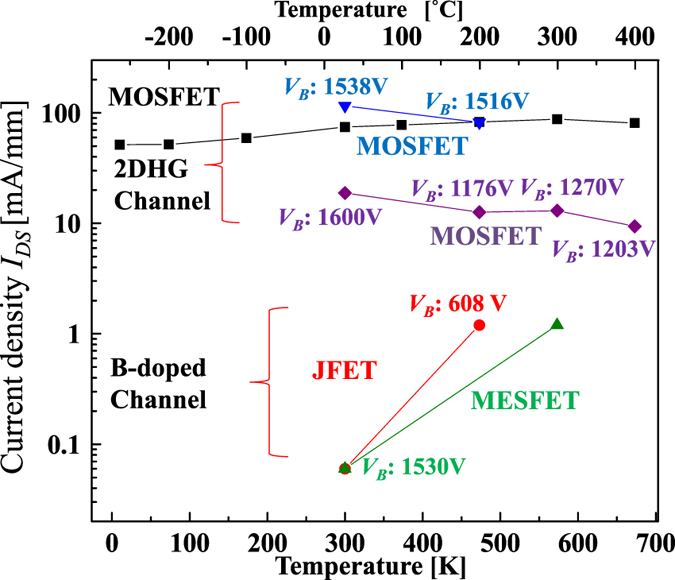
Temperature dependence of maximum drain current density (*I*_*DS*_) of diamond FETs. The current density is normalized with respect to the gate width. The temperature dependence of a MOSFET under drain source bias of 10 V and 50 V and that of a junction FET^25^ and a MESFET^23^ with a boron-doped channel at *V*_*DS*_ = 10 V are also shown.

**Table 1:** Comparison of maximum breakdown voltages* V_B Max_* and maximum drain current density *I_D Max_* at a particular *L_GD_* less than 20 μm in planar FETs of SiC, AlGaN/GaN, AlGaN/AlGaN, and diamond.

Wide Bandgap Planar FETs	*V*_*B MAX*_Breakdow Voltage	*L*_*GD*_ Gate Drain Distance	*V*_*B*_*/L*_*GD*_Breakdown Field	*I*_*D MAX*_Drain Current Density
SiC n-FET^19^	1600 V	20 μm	0.8 MV/cm	90 mA/mm
AlGaN/GaN^20^,^21^ n-FET	1500 V	15 μm	1.0 MV/cm	300-600 mA/mm
AlGaN/AlGaN^22^ n-FET	1700 V	10 μm	1.7 MV/cm	200 mA/mm
C-H Diamond p-FET	1700 V	16 μm	1.0 MV/cm	110 mA/mm

## References

[b1] Isberg J. . High carrier mobility in single-crystal plasma-deposited diamond. Science 297, 1670–1672 (2002). 12215638 10.1126/science.1074374

[b2] Ekimov E. . Superconductivity in diamond. Nature 428, 542–545 (2004). 15057827 10.1038/nature02449

[b3] Yokoya T. . Origin of the metallic properties of heavily boron-doped superconducting diamond. Nature 438, 647–650 (2005). 16319887 10.1038/nature04278

[b4] Nebel C. E. Chemistry. Surface-conducting diamond. Science 318, 1391–1392 (2007). 18048673 10.1126/science.1151314

[b5] Kawarada H. High-current metal oxide semiconductor field-effect transistors on H-terminated diamond surfaces and their high-frequency operation. Jpn. J. Appl. Phys. 51, 090111 (2012).

[b6] Mishra U. K., Parikh P. & Wu Y. AlGaN/GaN HEMTs-an overview of device operation and applications. PROCEEDINGS-IEEE 90, 1022–1031 (2002).

[b7] Kawarada H. Hydrogen-terminated diamond surfaces and interfaces. Surf. Sci. Rep. 26, 205–259 (1996).

[b8] Nebel C. . Hydrogen-induced transport properties of holes in diamond surface layers. Appl. Phys. Lett. 79, 4541–4543 (2001).

[b9] Strobel P., Riedel M., Ristein J. & Ley L. Surface transfer doping of diamond. Nature 430, 439–441 (2004). 15269764 10.1038/nature02751

[b10] Chakrapani V. . Charge transfer equilibria between diamond and an aqueous oxygen electrochemical redox couple. Science 318, 1424–1430 (2007). 18048683 10.1126/science.1148841

[b11] Kueck D., Schmidt A., Denisenko A. & Kohn E. Analysis of passivated diamond surface channel FET in dual-gate configuration—Localizing the surface acceptor. Diam. Relat. Mater. 19, 166–170 (2010).

[b12] Kasu M., Sato H. & Hirama K. Thermal stabilization of hole channel on H-terminated diamond surface by using atomic-layer-deposited Al_2_O_3_ overlayer and its electric properties. Appl. Phys. Express 5, 025701 (2012).

[b13] Hiraiwa A., Daicho A., Kurihara S., Yokoyama Y. & Kawarada H. Refractory two-dimensional hole gas on hydrogenated diamond surface, J. Appl. Phys. 112, 124504 (2012).

[b14] Werner F. & Schmidt J. Manipulating the negative fixed charge density at the c-Si/Al_2_O_3_ interface. Appl. Phys. Lett. 104, 091604 (2014).

[b15] Matsunaga K., Tanaka T., Yamamoto T. & Ikuhara Y. First-principles calculations of intrinsic defects in Al_2_O_3_. Phys. Rev. B 68, 085110 (2003).

[b16] Yang M. Y., Kamiya K. & Shiraishi K. Interstitial oxygen induced Fermi level pinning in the Al_2_O_3_-based high-k MISFET with heavy-doped n-type poly-Si gates. AIP Adv. 3, 102113 (2013).

[b17] Liu J., Liao M., Imura M. & Koide Y. Band offsets of Al_2_O_3_ and HfO_2_ oxides deposited by atomic layer deposition technique on hydrogenated diamond. Appl. Phys. Lett. 101, 252108 (2012).

[b18] Takahashi K., Imamura M., Hirama K. & Kasu M. Electronic states of NO_2_-exposed H-terminated diamond/Al_2_O_3_ heterointerface studied by synchrotron radiation photoemission and X-ray absorption spectroscopy. Appl. Phys. Lett. 104, 072101 (2014).

[b19] Noborio M., Suda J. & Kimoto T. 1580-V–40-double-RESURF MOSFETs on 4H-SiC. IEEE Electron Device Lett. 30, 831–833 (2009).

[b20] Lu B. & Palacios T. High Breakdown (>1500 V) AlGaN/GaN HEMTs by Substrate-Transfer Technology. IEEE Electron Device Lett. 31, 951–953 (2010).

[b21] Lee J. . High Breakdown Voltage (1590 V) AlGaN/GaN-on-Si HFETs with Optimized Dual Field Plates (CS MANTECH Conference, April 23rd-26th, 2012).

[b22] Nanjo T. . AlGaN channel HEMT with extremely high breakdown voltage. IEEE Trans. Electron Dev. 60, 1046–1053 (2013).

[b23] Umezawa H., Matsumoto T. & Shikata S. Diamond metal–semiconductor field-effect transistor with breakdown voltage over 1.5 kV. IEEE Electron Device Lett 35, 1112–1114 (2014).

[b24] Iwasaki T. . High-temperature operation of diamond junction field-effect transistors with lateral pn junctions. IEEE Electron Device Lett. 34, 1175–1177 (2013).

[b25] Iwasaki T. . 600 V diamond junction field-effect transistors operated at 200 °C. IEEE Electron Device Lett., 35, 241–243 (2014).

[b26] Hirama K., Sato H., Harada Y., Yamamoto H. & Kasu M. Diamond field-effect transistors with 1.3 A/mm drain current density by Al2O3 passivation layer. Japanese Journal of Applied Physics 51, 090112 (2012).

