# Supplementary material for: Durability-enhanced two-dimensional hole gas of C-H diamond surface for complementary power inverter applications
Source: Sci Rep. 2017 Feb 20;7:42368. doi: 10.1038/srep42368 (PMC5316979; doi:10.1038/srep42368)
Supplement: Supplementary Information [file srep42368-s1.doc]

Supplementary Materials

Title: Durability-enhanced two-dimensional hole gas of C-H diamond surface for complementary power inverter applications

Authors list:

Hiroshi Kawarada^1,2,3,*^, Tetsuya Yamada^1^, Dechen Xu^1^, Hidetoshi Tsuboi^1^, Yuya Kitabayashi^1^, Daisuke Matsumura^1^, Masanobu Shibata^1^, Takuya Kudo^1^, Masafumi Inaba^1^, Atsushi Hiraiwa^3^

^1^ Faculty of Science and Engineering, Waseda University,

3-4-1, Ohkubo, Shinjuku-ku, Tokyo 169-8555, Japan

^2^ The Kagami Memorial Laboratory for Materials Science and Technology, Waseda University, 2-8-26 Nishiwaseda, Shinjuku, Tokyo 169-0051, Japan

^3^Research Organization for Nano & Life Innovation, Waseda University,

513 Waseda-tsurumaki, Shinjuku, Tokyo 162-0041, Japan

*Tel: +81-3-5286-3391; E-mail: [kawarada@waseda.jp](mailto:kawarada@waseda.jp)

Figure S1. Schematic diagrams of (a) AlGaN/GaN and (b) ALD Al_2_O_3_/C-H diamond interfaces where a two-dimensional electron gas (2DEG) and a two-dimensional hole gas (2DHG) are produced, respectively. The 2DHG is ubiquitous where the C-H diamond surface is covered by a hole-inducing film, such as the ALD Al_2_O_3_ film used in this case.

Figure S2. Drain current vs. drain-source voltage (*I_D_–V_DS_*) characteristics of C-H diamond MOSFETs with 32-nm-thick and 200-nm-thick Al_2_O_3_ layers at −263°C (10 K), room temperature, and 400°C (673 K).

Figure S3. Drain-source current vs. gate-source voltage (*I_DS_*－*V_GS_*) characteristics of C-H diamond MOSFET with 32-nm-thick Al_2_O_3_ at various temperatures.

Figure S4. Schematic model of C-H diamond MOSFET with 2DHG used for device simulation. The negative fixed charge in Al_2_O_3_ is placed at the interface between C-H diamond and Al_2_O_3_, as proposed in Fig. 2a.

Figure S5 Off state *I_D_-V_DS_* characteristics showing blocking behavior of two C-H diamond MOSFETs with common channel width (25 μm) with *L_GD_* of 1 μm. Insets are on state *I_D_-V_DS_* characteristics up to *V_DS_* = 10 V in both FETs.

a b

Figure S6. (a) On-state and (b) off-state *I_D_-V_DS_* characteristics of C-H diamond MOSFETs with common channel width (25 μm) and *L_GD_* of 16 μm. The maximum drain current density per channel with *I_DS max_* is nearly 100mA/mm in the on-state. In the off-state, the breakdown voltage *V*_B_ is 1708 V.

Fig. S7. Fabrication process of C-H diamond MOSFET. (a) Homoepitaxial growth of undoped diamond on (001) high-pressure and high-temperature-synthesized diamond. (b) Source and drain formation by Au/Ti deposition. (c) Hydrogen termination, except at source and drain electrodes. (d) FET channel isolation by oxidation. (e) ALD Al_2_O_3_ layer formation for gate oxide and passivation. (f) Final MOSFET structure with Al gate on Al_2_O_3_.
